# Major cause of unprecedented Arctic warming in January 2016: Critical role of an Atlantic windstorm

**DOI:** 10.1038/srep40051

**Published:** 2017-01-04

**Authors:** Baek-Min Kim, Ja-Young Hong, Sang-Yoon Jun, Xiangdong Zhang, Hataek Kwon, Seong-Joong Kim, Joo-Hong Kim, Sang-Woo Kim, Hyun-Kyung Kim

**Affiliations:** 1 Korea Polar Research Institute, Incheon, South Korea; 2 University of Alaska, Fairbanks, USA; 3 Seoul National University, Seoul, South Korea; 4 Korea Meteorological Administration, Seoul, South Korea

## Abstract

In January 2016, the Arctic experienced an extremely anomalous warming event after an extraordinary increase in air temperature at the end of 2015. During this event, a strong intrusion of warm and moist air and an increase in downward longwave radiation, as well as a loss of sea ice in the Barents and Kara seas, were observed. Observational analyses revealed that the abrupt warming was triggered by the entry of a strong Atlantic windstorm into the Arctic in late December 2015, which brought enormous moist and warm air masses to the Arctic. Although the storm terminated at the eastern coast of Greenland in late December, it was followed by a prolonged blocking period in early 2016 that sustained the extreme Arctic warming. Numerical experiments indicate that the warming effect of sea ice loss and associated upward turbulent heat fluxes are relatively minor in this event. This result suggests the importance of the synoptically driven warm and moist air intrusion into the Arctic as a primary contributing factor of this extreme Arctic warming event.

The Arctic has been warming in recent decades in an amplified manner and this warming is accompanied by an unprecedented reduction of sea ice and high-latitude snow cover, which creates a positive feedback loop^1^. The influence of Arctic warming, however, is not confined to the Arctic region. Recent studies have provided firm evidence of the substantial remote influence of the warm Arctic. In particular, the below-normal sea ice condition over the Atlantic Arctic sector, such as the Barents and Kara seas, is known to be linked to the Eurasian continental cooling in recent consecutive cold winters^2^,^3^,^4^,^5^,^6^,^7^.

Despite the importance of the Arctic state for the adjacent mid-latitudes, the major cause of Arctic warming remains a controversial issue. Several important factors of Arctic warming have been suggested, including the following: 1) surface reflectivity of snow and ice^8^, 2) oceanic heat loss by surface turbulent heat fluxes^9^, 3) incoming longwave radiation emitted by water vapor and clouds^10^,^11^, 4) surface thermal inversion^12^, 5) atmospheric lapse-rate^13^, and 6) poleward atmospheric energy transport by moisture intrusion^14^,^15^,^16^,^17^. These local and remote factors are strongly coupled and should collectively contribute to Arctic warming.

The warming factors interplay with the multi-scale internal variability from synoptic-scale to low-frequency. Either a single storm event or a series of events associated with low-frequency variability, such as blocking flows and atmospheric teleconnection patterns^18^,^19^, can stir the adjacent ocean through surface turbulent processes and bring warm and humid air from lower latitudes to higher latitudes. This occurrence weakens the surface temperature inversion of the Arctic, reduces the thermodynamic stability by changes in the lapse rate, and increases the incoming longwave radiation by moisture and clouds. Therefore, multiple processes of Arctic warming can be investigated by analyzing storms that enter the Arctic.

In the winter season of 2015–16, an extraordinary increase in the Arctic surface air temperature (SAT) was observed after an extreme Atlantic windstorm entered the Arctic Circle in late December 2015. This storm, which was named ‘Frank’ by the U.K. Met Office, recorded a minimum central pressure of 928 hPa on 30 December 2015^20^. Maximum Arctic-mean (north of 65°N) SAT anomalies as high as 7° to 8 °C were recorded on 1–2 January after the storm underwent cyclolysis along the eastern coast of Greenland. The anomalous warm temperature was maintained during the entire month of January, which produced a record anomalous Arctic-mean temperature^21^. In particular, the SAT locally increased to the extreme value of approximately 30 °C higher than the normal SAT in January over the Eurasian Arctic sector. This event appeared to be super-extreme in the sense that no warming event had developed as rapidly as this event or maintained for such a long period (Supplementary Table S1). This case is ‘unprecedented’ for the available period of modern data and provides an invaluable opportunity to scrutinize a slice of extreme warming in the Arctic.

In this study, we show that the extreme storm event caused a large heat and moisture intrusion into the Arctic and triggered the subsequent operation of multiple feedback mechanisms, resulting in the record Arctic warming event.

## Results

### Arctic warming in January 2016

**Figure 1:**
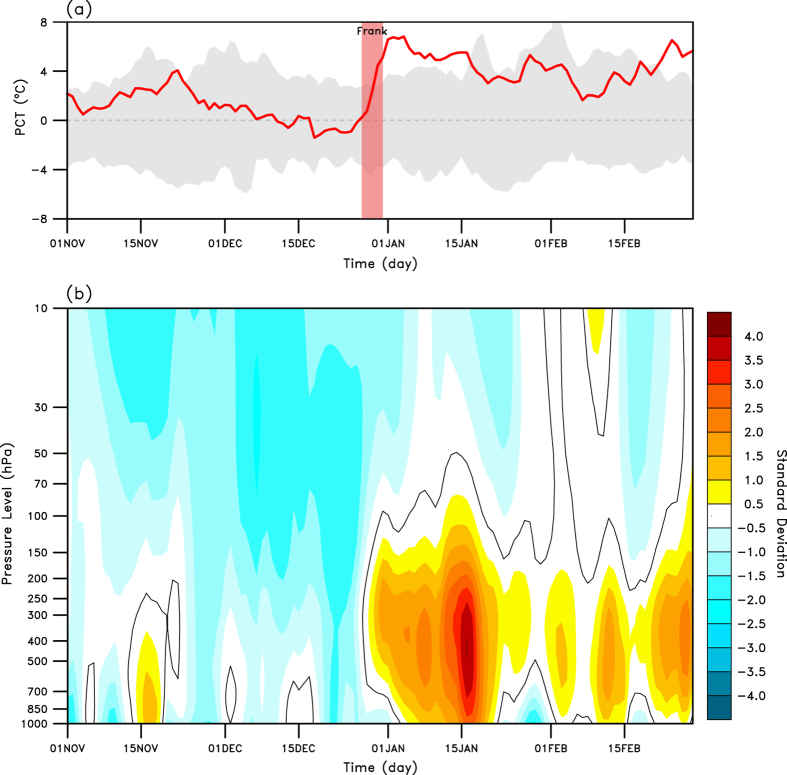
Observed anomalies of SAT and normalized geopotential height over the polar cap. (**a**) Polar cap (north of 65°N) SAT anomalies (red line) and (**b**) normalized polar cap geopotential height anomalies (shading) at 32 pressure levels from 1 November 2015 to 28 February 2016. In (**a**), the range of historical daily SAT anomalies are shaded using the data from 1979–80 to 2014–15 and the transparent red bar indicates the lifetime of Storm Frank. The black solid lines in (**b**) show zero anomalies.

First, we reconfirm that the Arctic warming event that occurred in January 2016 was record-breaking in multiple aspects (Fig. 1a). The Arctic-wide temperature increase, which was measured by the polar cap temperature anomaly (PCT; refer to Methods) at the beginning of the event was abruptly initiated, as indicated by an increase of approximately 6 °C in three days. This increase was a historical record; the highest daily temperature anomaly corresponds to a level beyond the 3.5 standard deviation level. The PCT retained the values at a record level for more than 15 consecutive days. At the beginning of the record Arctic warming event, the extreme Atlantic storm Frank entered the Arctic Circle. Table 1 lists the top five strongest windstorms that have occurred in the North Atlantic since 1958. According to the table, the lowest central pressure of 913 hPa was recorded for the storm that occurred in January 1993. Storm Frank ranked as the fourth strongest storm when the lowest central pressure reached 928 hPa.

A sudden shift in the large-scale weather regime in the Northern Hemisphere occurred after the decay of Storm Frank (Fig. 1b). The time–height section of the normalized polar cap height anomaly (PCH) is often regarded as an indicator of large-scale weather regimes because it is well correlated with the annular mode indices in both hemispheres^22^ (refer to Methods). Note that the PCH was generally negative on every vertical level of the troposphere and stratosphere until the end of 2015, which indicates that the weather was under the control of the positive Northern Hemisphere annular mode (NAM)^23^ in early winter of 2015–16, during which the weather systems move fast and blocking activities are generally suppressed. The weather regime significantly shifted to the negative NAM phase on 2 January. Typical SAT conditions of the negative NAM were observed in many places of the Northern Hemisphere; for example, the anomalous cooler condition across central Asia and China and the very warm condition over parts of northern and eastern Russia with some regions experiencing record warm temperatures^24^. On 22–24 January, a severe winter storm hit the northeastern United States, and new snowfall records were set for several cities, including New York^24^. These events are weather conditions that are usually embedded in the negative NAM^23^ From a hemispheric circulation perspective, winter 2015–16 (November 2015 to February 2016) can be featured by a dramatic alteration of weather regime (Fig. 1b). The shift in weather regime, the entry of a strong Atlantic windstorm into the Arctic and the initiation of extreme Arctic warming simultaneously occurred. As discussed in the next section, the co-occurrence of these events was not a coincidence but a physical linkage.

### Storm Frank and Changes in Arctic Circulation

**Figure 2:**
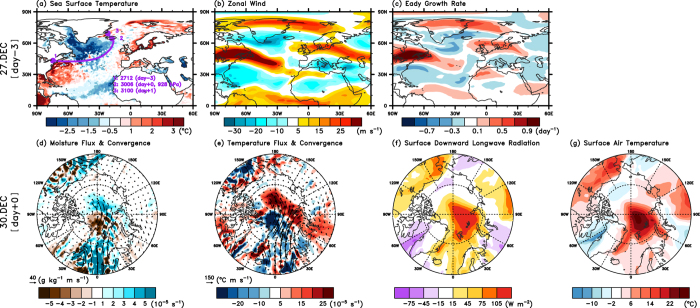
Anomalous atmospheric and SST conditions for the initial date (day −3) and the peak date (day 0) of Storm Frank. (**a**) SST (shading) and storm trajectory (purple line), (**b**) 300-hPa zonal wind, (**c**) Eady growth rate between 200 and 850 hPa, (**d**) 850-hPa moisture flux (arrows) and its convergence (shading), (**e**) 850-hPa temperature flux (arrows) and its convergence (shading), (**f**) surface downward longwave radiation (downward positive), and (**g**) SAT. The NCAR Command Language (NCL) with version 6.3.0 (http://dx.doi.org/10.5065/D6WD3XH5) was used to generate the maps in this figure.

We describe the life cycle of Storm Frank. An extratropical depression became a storm on 27 December while it was located near the east coast of North America (day −3 in Fig. 2a). The storm traversed the North Atlantic, recurved northward towards the Arctic on 29 December, and rapidly intensified to a powerful storm with a minimum central surface pressure of 928 hPa on 30 December. It subsequently merged into a preexisting stationary depression over the Denmark Strait and gradually weakened. On 27 December, the storm was initiated over the east coast of North America, where the Atlantic sea surface temperature (SST) gradient was the largest (Fig. 2a). On the date of initiation, we also note that the strong 300-hPa zonal wind anomaly passed over the storm genesis region (Fig. 2b). Consistent with this pattern of the upper-level zonal wind anomaly, the Eady growth rate (refer to Methods) also exhibited maxima over the genesis region, which indicates enhanced baroclinic instability (Fig. 2c). Note that the state of the NAM is strongly related to the frequency, intensity, and tracks of Atlantic windstorms^25^. The strong storms are usually embedded in the strong large-scale westerly flow^26^, which implies that the positive NAM favors frequent storms with strong intensity in the North Atlantic compared with the negative NAM. As revealed in Fig. 2c, this finding is attributed to the positive growth rate along the region of enhanced westerly jets during the positive NAM period (day −3). Therefore, the anomalous SSTs (Fig. 2a) and large-scale flows (Fig. 2b) provided favorable conditions for cyclogenesis in the eastern coastal region of North America.

On the peak date of Storm Frank (day 0 in Fig. 2), the most pronounced features related to the storm were anomalous poleward low-level moisture and heat fluxes from the North Atlantic (Fig. 2d and e). Associated with the tremendous influx of moisture and heat to the Arctic, the downward longwave radiation was substantially reinforced (Fig. 2f) and the SAT was extremely warm (Fig. 2g). Although another region of Arctic warming existed over northwestern America (60°–70°N, 110°–170°W), the warming in the Atlantic Arctic sector associated with Storm Frank dominated the pan-Arctic temperature increase. Note that the general pattern of the SAT anomalies is very similar to the general pattern of the downward longwave radiation anomalies. The spatial correlation coefficient between the two variables is 0.87. The detailed daily evolution of the surface cyclone and associated changes in the moisture flux are depicted in Supplementary Fig. S1 from 25 December 2015 to 2 January 2016. The daily evolution of the SAT anomalies is also shown in Supplementary Fig. S2.

**Figure 3:**
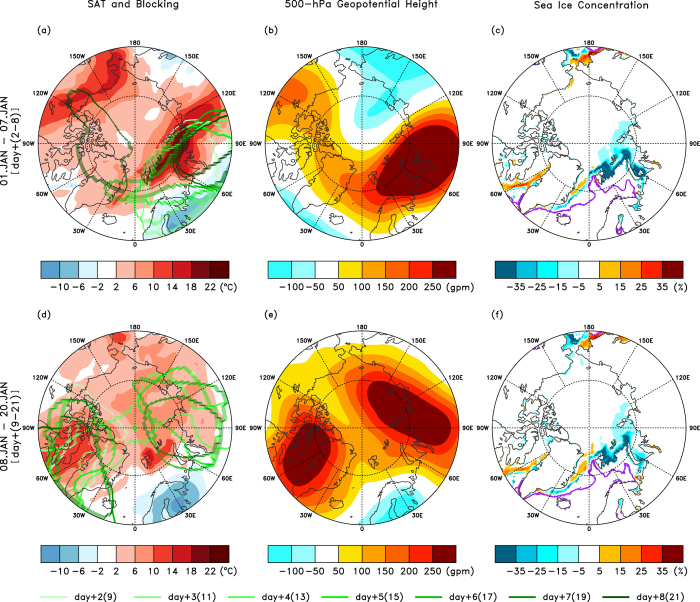
Atmospheric and sea ice responses after the termination of Storm Frank. (**a,d**) averaged SAT anomalies (shading) and individual blocking areas (coloured closed curves; refer to Methods), (**b,e**) averaged 500-hPa geopotential height anomalies, and (**c,f**) sea ice concentration anomalies. Upper rows and lower rows contain the first seven days (1–7 January) and the next 13 days (8–20 January), respectively. In (**a**) and (**d**), the coloured curves denote the timing of the blocking event with brighter colours for earlier dates and darker colours for later dates. In (**c**) and (**f**), the purple coloured lines depict climatological sea ice edge boundaries (sea ice concentration of 15%). As with previous figures, 30 December is defined as day 0. The NCAR Command Language (NCL) with version 6.3.0 (http://dx.doi.org/10.5065/D6WD3XH5) was used to generate the maps in this figure.

As depicted in Fig. 1, the PCT abruptly increased and the PCH also changed sign to positive, i.e., anomalously high over the Arctic, in the troposphere during the entry of Storm Frank into the Arctic. Both changes in temperature and circulation over the Arctic occurred abruptly and the anomalous positive values were maintained for more than one month. Figure 3 summarizes key environmental features as an aftermath of the extreme storm event. For the first seven days (1 to 7 January), the anomalous SAT was prominent in the northern Barents and Kara seas towards central Siberia, with a maximum local increase of up to 23 °C (Fig. 3a). We also observe an apparent warm anomaly over Alaska and northern Canada although the degree of warming was relatively smaller than the degree of warming in the Atlantic and Eurasian sectors. These two warming centers contributed to the PCH depicted in Fig. 1b. Although not shown, the warming over the Pacific sector was also contributed by a storm event that occurred over the North Pacific Ocean.

Among the notable circulation features during the anomalous warm event, multiple blocking events occurred during this period (refer to Methods). A large contrast in the blocking activity before and after the storm event was noted. Before the decaying date of Storm Frank (i.e., 31 December 2015), the blocking detection algorithm does not detect any single blocking event in the Northern Hemisphere for the period from 25 December to 31 December 2015 but detects a blocking event after 1 January 2016 over Scandinavia and northwestern Russia (Supplementary Fig. S3). Although positive geopotential height anomalies occurred over Europe while Storm Frank was in his earlier stage over the eastern coast of North America (25 to 28 December), the algorithm does not detect them as blocking areas because these anomalies did not pass the thresholds. Meanwhile, it should be noted that the preexisting low-frequency waves influenced the storm trajectory. However, Supplementary Fig. S1 demonstrates that the intensive cyclonic circulation embedded in the low-frequency waves served an essential role in initiating the abrupt Arctic warming event on 28 December (Supplementary Fig. S2).

During the first week after the storm termination, a blocking event was initiated over the European region (light green curves in Fig. 3a). The occurrence of the European blocking (or Scandinavian blocking) after the breaking of an Atlantic windstorm is not a rare event but has been discussed in previous studies^27^,^28^. In our case, the northeastward intrusion of low potential vorticity at 350 K was observed over western Europe while Storm Frank was traversing the ocean between Greenland and Europe during 29–31 December 2015 (Supplementary Fig. S4). This feature is a distinct feature of the anticyclonic wave breaking of synoptic waves^27^. The average pattern of the mid-tropospheric circulation anomaly also reflects the blocking feature over Scandinavia and northwestern Russia (Fig. 3b). In this situation, the anticyclonic flows induced by the blocking helped the warm maritime air to flow into the Arctic.

During the next two weeks (8 to 20 January), notable changes in circulation occurred. As depicted in Fig. 3d, two major blocking activities for this period occurred. The blocking activity over the European region slightly moved eastward and changed its axis of orientation northeastward and the blocking activity around Greenland enhanced considerably (Fig. 3e). The two blocking highs in the troposphere of the Arctic Circle contributed to the large increase of the PCH in mid-January, as depicted in Fig. 1b. Note that the SAT anomalies depicted in Fig. 3a and d coincided with the geopotential height anomalies, which indicates that the warming was largely sustained by the circulation anomalies. However, the enhancement of the Greenland blocking and the eastward migration of the European blocking reversed the poleward transport of moist and warm air from the North Atlantic Ocean. Thus, the SAT anomaly became smaller though remained positive (Fig. 1a).

In addition to the extreme Arctic warming, the sea ice concentration along the ice margin of the Barents and Kara seas decreased substantially (Fig. 3c and f). Compared with the first week, the sea ice slightly recovered during 8–20 January due to the changed circulation feature but remained lower than normal. The sea ice reduction caused an increase in the ocean heat release by upward turbulent heat flux anomalies over 70 W m^−2^ (with a maximum heat flux anomaly of 141 W m^−2^) along the ice margin for an average of 20 days (Supplementary Fig. S5).

Based on the observational features, the role of the moist and warm air intrusion for the extreme Arctic warming is distinct but an additional contribution of bottom heating by turbulent heat fluxes cannot be eliminated for the reduced sea ice state during this event. Two competing heating sources have been a popular topic for understanding time-mean Arctic warming in the previous literature^16^,^17^,^29^,^30^,^31^. A number of studies attributed the bottom-heavy warming characteristics of Arctic amplification to increased upward turbulent heat fluxes by sea ice loss^29^,^30^. Recently, however, an alternative physical mechanism has been proposed to explain the bottom-heavy warming profile with the synoptically driven moisture intrusion from the lower latitudes and the associated increase in downward longwave radiation^31^. The horizontal moisture and heat transport warms the surface by increased longwave heating and weakens the inversion strength, which is consistent with recent observational and modelling studies^13^,^14^,^15^. With this mechanism, the bottom-heavy warming in the Arctic can be explained even in the absence of sea ice loss.

### Model simulation results

**Figure 4:**
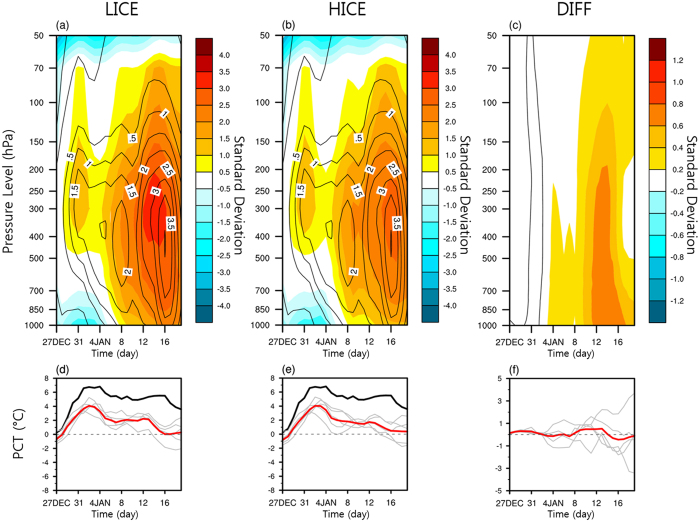
Model-simulated anomalies of SAT and normalized geopotential height over the polar cap. Ensemble mean of (**a,b**) the normalized polar cap geopotential height anomalies (shading) and (**d,e**) the polar cap SAT anomalies (red line) from 27 December 2015 through 23 January 2016 from the Polar WRF ensemble experiments. The corresponding variables from the JRA55 data are overlaid with black contours for the geopotential height anomalies and a black line for the polar cap SAT anomalies. The geopotential height anomalies in (**a**) and (**b**) are normalized by the standard deviation for the period of 1981–2010. The left column and middle column correspond to “LICE” and “HICE”, respectively, and the right column (**c,f**) contains their differences (LICE minus HICE). The black solid lines in (**c**) show the zero anomalies. The gray lines for the bottom panels indicate the results from individual ensemble members.

For the extreme Arctic warming event in January 2016, both the strong moist and warm air intrusion from the North Atlantic and the sea ice loss in the Barents and Kara seas were observed, which provides an invaluable opportunity to examine the relative importance of individual factors to the extreme warming event. To assess the relative importance of the sea ice loss, compared to the horizontal transport, we design two sets of ensemble numerical experiments, each composed of a low sea ice condition (LICE) and a high sea ice condition (HICE) (refer to Methods). With the exception of the sea ice boundary condition and associated SST correction, where the sea ice cover is modified^32^, corresponding members of both sets used the same initial and lateral boundary conditions. Therefore, the ensemble-mean difference between the two sets can be interpreted as an impact of the different sea ice conditions. To compare the model results with the observations in a simple framework, we calculate the ensemble-mean PCH (Fig. 4a,b) and PCT (Fig. 4d,e) using the model results. A comparison of the two experiments reveals that the Arctic warming event is comparably simulated although weaker in magnitude than the observed event, despite the substantial difference in the lower boundary condition over the sea ice-modified area. The ensemble-mean differences between HICE and LICE seen in Fig. 4c and f are relatively small compared to internal variability, and according to a two-tailed Student’s *t*-test, none of them is significant at the 10% level. Although it seems to exhibit an ensemble-mean difference with a bottom-heavy profile in mid-January (Fig. 4c), the difference is not statistically meaningful due to a large spread of the ensemble members. Therefore, the model results suggest the importance of the synoptically driven horizontal transport of moist and warm air as a dominating factor for the extreme Arctic warming event in mid-January.

## Summary and Discussion

An extreme Arctic warming event occurred in the winter season of 2015–16. Several special characteristics distinguish this event from similar events. The initial Arctic-wide temperature increase rapidly exceeded 6 °C in the last four days of 2015, and the extremely warm temperature above two standard deviations of the normalized PCT was maintained for almost 40 days (Supplementary Table S1). Both characteristics are unprecedented in the Arctic, at least for the available data period.

First, we paid attention to the initial explosive development of this event and found the role of a great Atlantic windstorm ‘Frank’ that entered the Arctic. The energy source that drove this storm to such an extreme was a combination of an initial pulse of heat that was picked up when it passed over very warm Gulf Stream waters and the strong upper-tropospheric zonal winds along the jet stream. The latter feature is typical of the positive NAM. As the storm approached the Arctic, the Arctic shifted to the weather regime opaque to longwave radiation^31^ due to the large poleward intrusion of moisture and its convergence in the Arctic Ocean (Supplementary Fig. S6). Therefore, the extreme windstorm that entered the Arctic Ocean was a major factor of initiating the extreme Arctic warming event. After the extreme storm initiated the event, large downward longwave radiation triggered several other mechanisms that helped to sustain the warming. A positive lapse rate feedback was activated, and enhanced vertical mixing eroded the surface inversion to reduce longwave cooling^13^. In addition to these thermodynamically driven processes, the sustaining mechanism also includes the generation of dynamically sustained blocking flows in the Eurasia and Greenland regions. In the blocking area, the warm advection and thickened geopotential height help to maintain warm temperatures at the surface^33^,^34^. Therefore, both thermodynamic and dynamic processes worked to maintain the Arctic warming. A recent study found that planetary waves serve a larger role for Arctic temperature anomalies than synoptic cyclones^17^. Our case study does not contradict this study, in the context that the blocking flows served a central role in sustaining the Arctic warming in January 2016.

The sea ice concentration in the Barents and Kara seas was also substantially reduced during the warming event. However, quantification of the cause of sea ice reduction is difficult in this case, because both the atmospheric radiative forcing and the oceanic warm water intrusion were combined with the mechanical forcing by the strong windstorm. Quantifying the role of upward surface turbulent heat fluxes over the area of sea ice loss for the Arctic warming is more relevant. The two sets of regional model experiments—experiments with an actual daily variation of sea ice concentration during the event and experiments with sea ice concentration artificially increased to the level of the early 1980 s—indicate that the extreme Arctic warming event is comparably simulated in both experiment sets. This finding suggests the atmospheric horizontal transport of heat and moisture to the Arctic to be a dominating factor at least for the extreme Arctic warming in January 2016.

Although this single event cannot be directly linked to the concept of Arctic amplification, this extremely anomalous event in the Arctic may have lingering effects on slow-varying sea ice and ocean, which may contribute to Arctic amplification of a recent warming trend. Yet, it is not known whether this event is an archetype of what has been happening recently under the Arctic warming trend. We should investigate whether triggering a pulse of Arctic warming by an intense synoptic storm that enters the Arctic and the subsequent development of planetary-scale blocking flows, as in this case, have contributed to Arctic warming. This advanced topic on a climatic time scale requires additional investigation.

## Methods

### Data and definitions of key variables

The meteorological variables are obtained from the Japanese 55-year Reanalysis data^35^. The high-resolution National Oceanic and Atmospheric Administration (NOAA) optimum interpolation SST version 2 dataset (OISST v2)^36^ is used for the sea ice cover and SST. These datasets are used for both the observational analyses and the lower boundary conditions of the model experiments. The period of the main analyses for the 2015–16 winter event encompasses 1 November 2015 to 28 February 2016. The anomalies are calculated based on the 30-yr climatology for the period of 1981–2010. The National Centers for Environmental Prediction (NCEP) Final Operational Global Analysis (FNL) data^37^ are obtained to initialize the model and prescribe the lateral boundary condition.

The Arctic warming event is quantitatively measured using the PCT, which is defined as the SAT anomaly averaged inside the Arctic Circle (north of 65°N). An indicator of large-scale weather regimes is defined by the PCH, which is obtained by averaging geopotential height anomalies over the area north of 65°N and normalizing the averaged anomaly by the standard deviation for each of the pressure levels. By definition, it is negatively correlated with the NAM^22^.

The Eady growth rate is calculated using


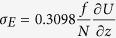


where *f* is the Coriolis parameter, *N* is the Brunt-Vaisala frequency, *U* is the zonal wind, and *z* is the height^38^. The mid-tropospheric Eady growth rate (200 minus 850 hPa) is evaluated in this study.

### Blocking detection algorithm

Blocking is detected using a recently developed algorithm^39^, which is a hybrid algorithm that combines two widely used indices: the Dole and Gordon type index^40^ and the Tibaldi and Molteni type index^41^. This method searches for persistent contiguous areas of geopotential height anomalies, as in the Dole and Gordon type index, which occurs in conjunction with the meridional reversals of the 500-hPa geopotential height as in the Tibaldi and Molteni type index. The threshold values that are necessary to detect the blocking event are as follows:

1. The amplitude threshold is set to 1.5 standard deviations of the geopotential height anomalies over 30°–90°N for a three-month period centered at a given month.
2. The spatial-scale threshold for closed contours, which satisfies the minimum amplitude threshold for geopotential height anomalies, is set to 2.5 × 10^6^ km^2^.
3. The overlap threshold is set to 50% of the area overlap in blocking areas within two days, which detects only quasi-stationary systems.
4. The duration criterion is set to five consecutive days.

### Experimental design

To investigate the sensitivity of Arctic warming to the sea ice conditions, two sets of ensemble numerical experiments are performed using the Polar Weather Research and Forecast model (WRF)^42^ with a 36 km horizontal resolution that encompasses the entire Arctic and adjacent North Atlantic sector (Supplementary Fig. S7). The NCEP FNL data are used to provide the initial and lateral boundary conditions for all experiments. Each experiment set consists of five ensemble members, each of which are configured with the initial time shifted by one day from 00 UTC 23 to 00 UTC 27 December 2015. All experiments end on 20 January 2016. Although this type of ensemble generation is not sufficient to sample the entire range of possibilities, we adopt this method from the concept of ensemble generation in the NCEP operational climate forecast system^43^. Of course, shifting by one day does not make a large difference near the initial time; however, this choice is intentional because all experiments must simulate the extreme Atlantic windstorm in late December in a similar fashion. With the constraint of the similar Atlantic windstorm that enters the Arctic at the initial period, the sensitivity to reduced sea ice can be fairly evaluated for the follow-up warming event.

The only difference between the two sets of experiments is a prescribed surface boundary condition for the entire simulation period. The control experiments (LICE) utilize daily varying gridded data of the SST and sea ice cover. For the sensitivity experiments (HICE), we hypothetically generate reconstructed daily sea ice cover over the domain of the Barents and Kara seas (67°–82°N, 10°–80°E; Supplementary Fig. S8), which preserves the day-to-day variability of the 2015–16 event period as much as possible, with the exception of having the average condition in the early 1980 s over the domain. This method aims to produce smoothly varying high sea ice concentration in space and time. To make this sea ice condition, we calculate the time-reversed increasing trends of sea ice cover in January of past decades (2013–14 to 1981–82) for all grid points over the domain of the Barents and Kara seas. The reconstruction factors are generated by multiplying the time-reversed trends in the individual grids by the total number of years (i.e., 33), which are added to the daily sea ice concentration of the corresponding grid point from 23 December 2015 to 20 January 2016. If the reconstructed sea ice concentration is larger than one at a grid point, the point is set to one. Supplementary Fig. S8 displays the mean spatial patterns of sea ice concentration averaged for 1–20 January for LICE and HICE, respectively, with their area-mean temporal changes. The SSTs over the sea ice-increased grid points were also adjusted to fit the changed sea ice fraction by the statistical relationship between sea ice concentration and SST^32^. The statistical relationship was constructed by the third-order polynomial fit (*ŷ* = **a **+ **b***x*** **+ **c***x*^2^** **+ **d***x*^3^; where *x* is the sea ice concentration and *ŷ* is the fitted SST.) for January using the OISST v2 for the period of 1982–2000^32^.

## Additional Information

**How to cite this article:** Kim, B.-M. *et al*. Major cause of unprecedented Arctic warming in January 2016: Critical role of an Atlantic windstorm. *Sci. Rep.* **7**, 40051; doi: 10.1038/srep40051 (2017).

**Publisher's note:** Springer Nature remains neutral with regard to jurisdictional claims in published maps and institutional affiliations.

## Supplementary Material

Supplementary Information

## Figures and Tables

**Table 1:** List of the top five strongest windstorms that occurred in the North Atlantic during the winter seasons of 1979–2015.

Rank	Name	Date	Minimum central pressure (hPa)
1	Braer	11 January 1993	913^44^
2	Noname	15 December 1986	916^45^
3	Dirk	24 December 2013	927^46^
4	Frank	30 December 2015	928^47^
5	Vivian	26 February 1990	940^48^
