# Supplementary material for: Major cause of unprecedented Arctic warming in January 2016: Critical role of an Atlantic windstorm
Source: Sci Rep. 2017 Jan 4;7:40051. doi: 10.1038/srep40051 (PMC5209705; doi:10.1038/srep40051)
Supplement: Supplementary Information [file srep40051-s1.pdf]

Table S1. Top four extreme Arctic warming events for 58 winter seasons of 1958-2015. Normalized daily PCT (refer to Methods) is used to define extreme Arctic warming events. The Arctic warming events are identified by the consecutive period during which the normalized PCT exceeds one standard deviation. The extreme Arctic warming events are identified by the consecutive period during which the normalized PCT exceeds two standard deviations.

Rank	Start	End	Duration (days)
1	29 December 2015	6 February 2016	40
2	25 January 2014	17 February 2014	24
3	30 January 2012	20 February 2012	22
4	2 January 1977	20 January 1977	19
	28 November 2007	16 December 2007	

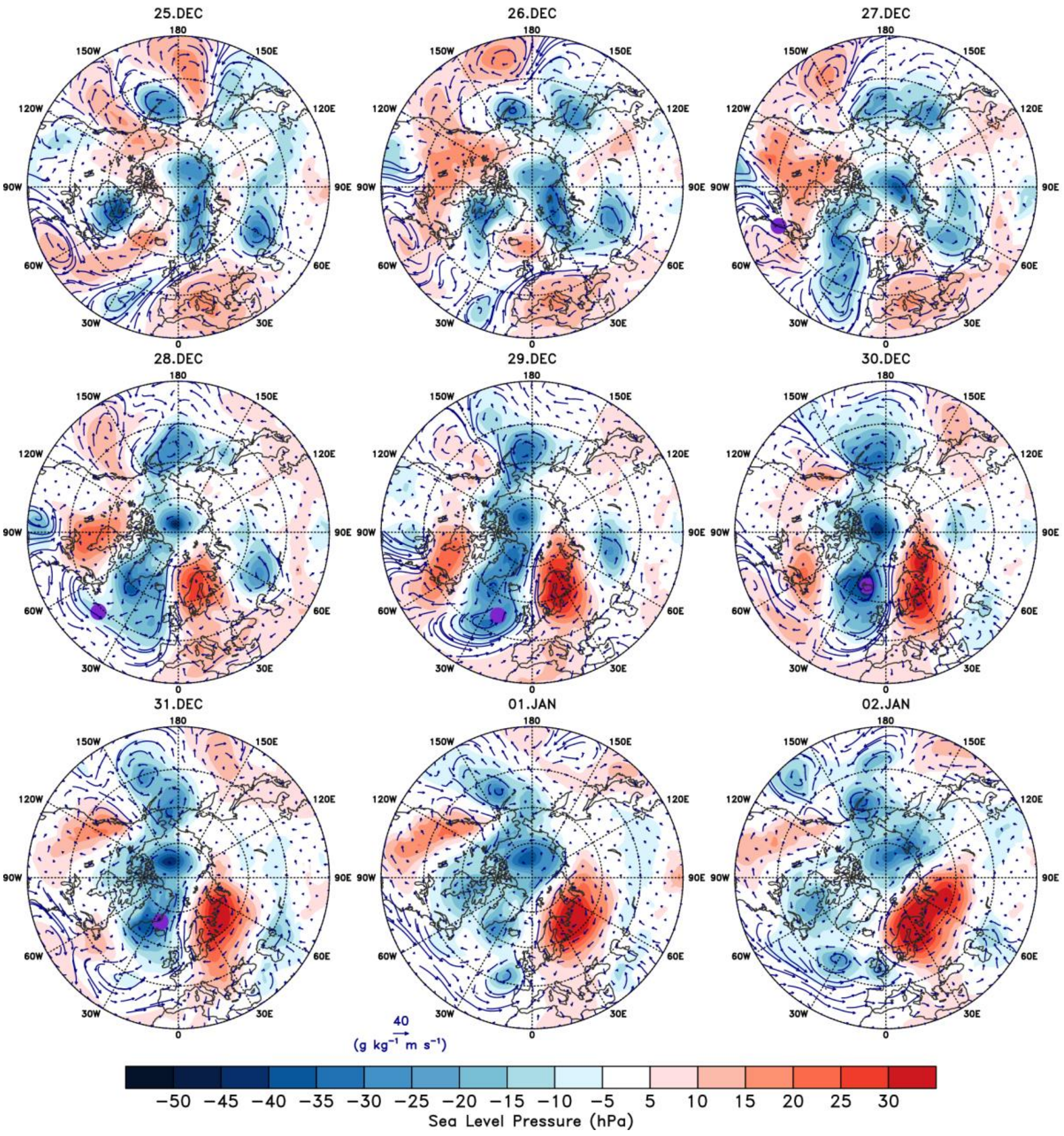

Figure S1. Anomalies of the sea level pressure (shading) and the 850-hPa moisture flux (arrows) from 25 December 2015 to 2 January 2016. The purple dot indicates the daily averaged center of Storm Frank. The NCAR Command Language (NCL) with version 6.3.0 (http://dx.doi.org/10.5065/D6WD3XH5) was used to generate the maps in this figure.

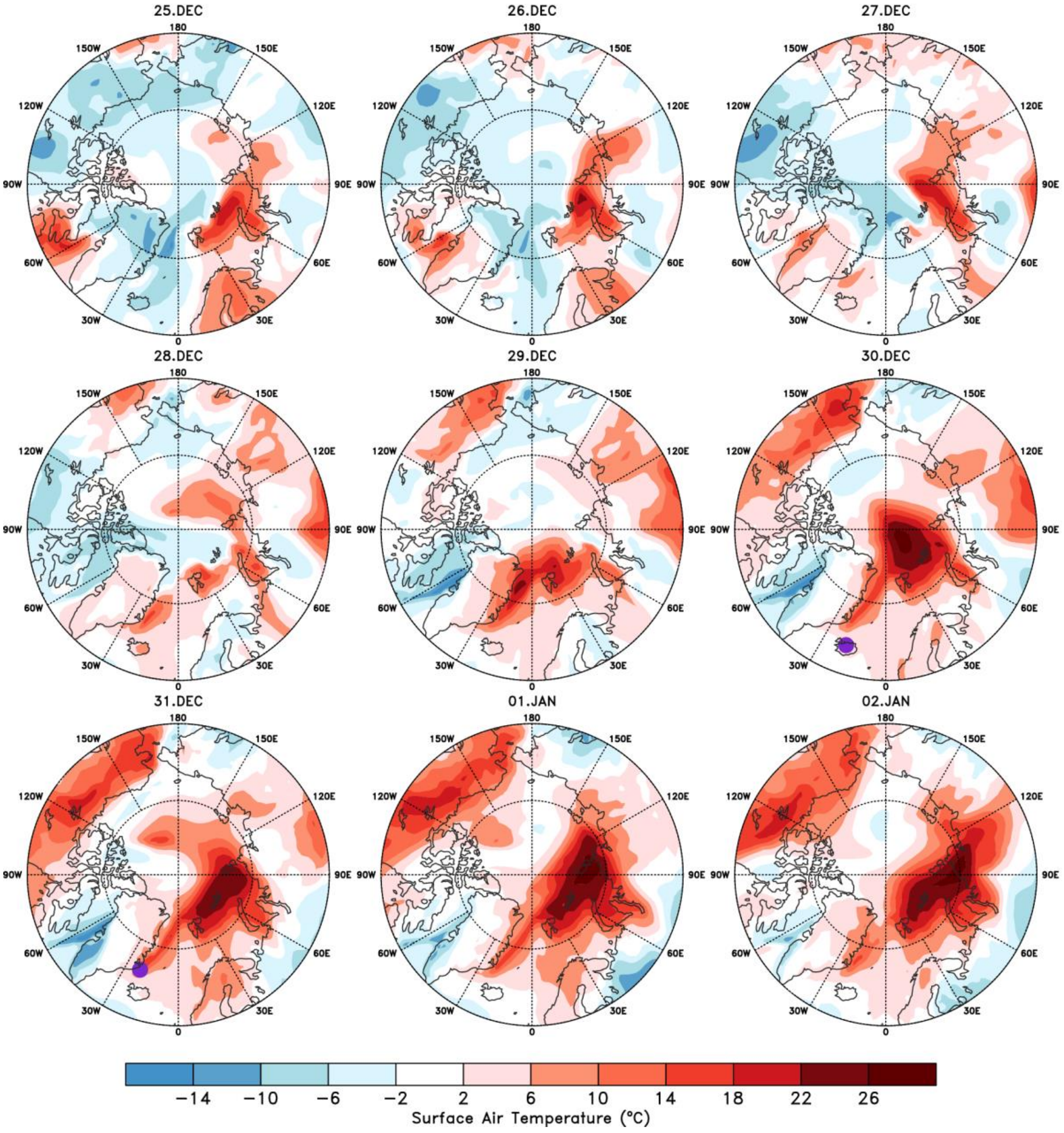

Figure S2. Same as Fig. S1, except for the SAT anomalies confined to the Arctic domain (north of 60°N). The NCAR Command Language (NCL) with version 6.3.0 (http://dx.doi.org/10.5065/D6WD3XH5) was used to generate the maps in this figure.

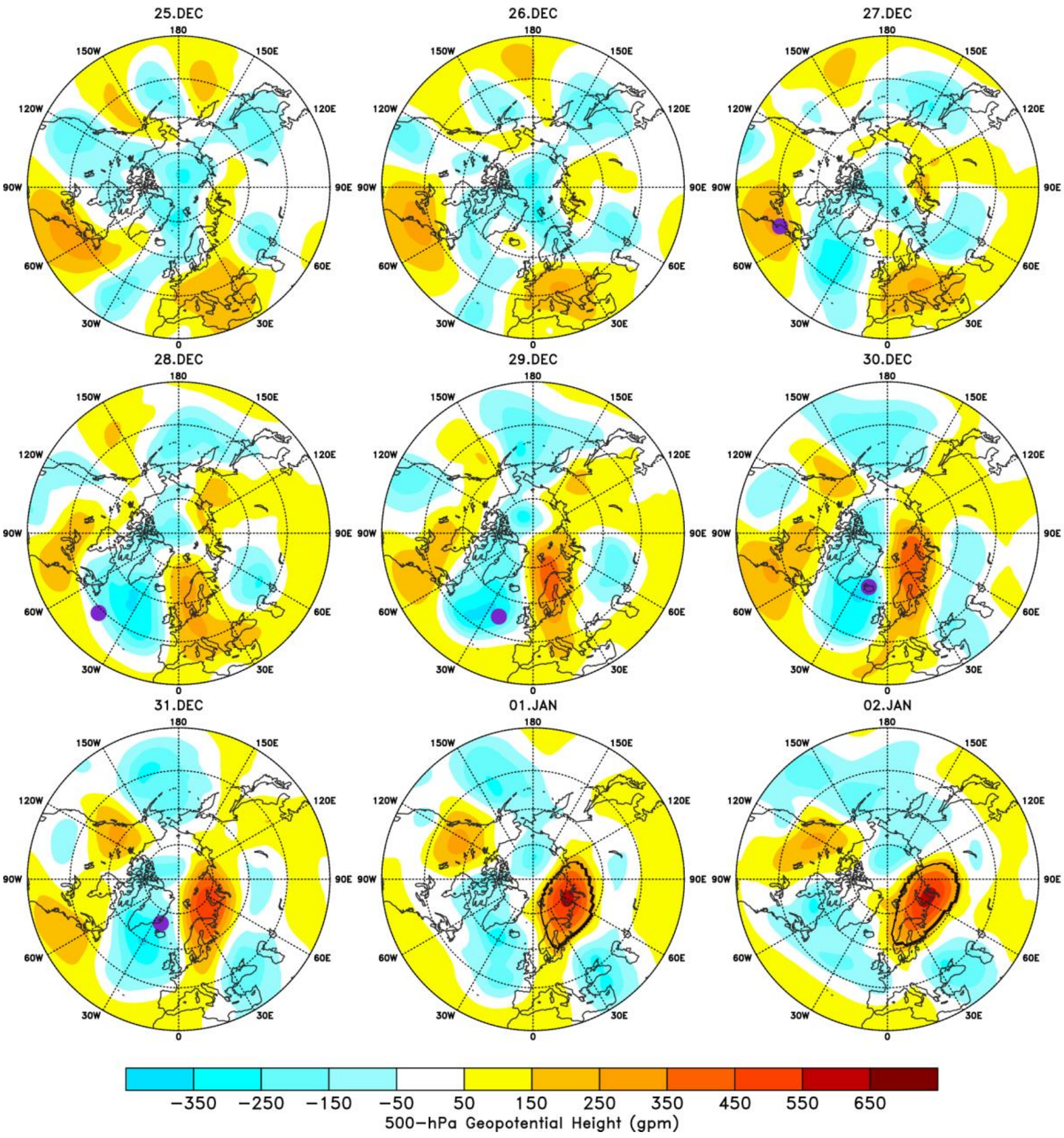

Figure S3. Same as Fig. S1, except for the 500-hPa geopotential height anomalies (shading) and detected blocking area (black closed contour). The NCAR Command Language (NCL) with version 6.3.0 (http://dx.doi.org/10.5065/D6WD3XH5) was used to generate the maps in this figure.

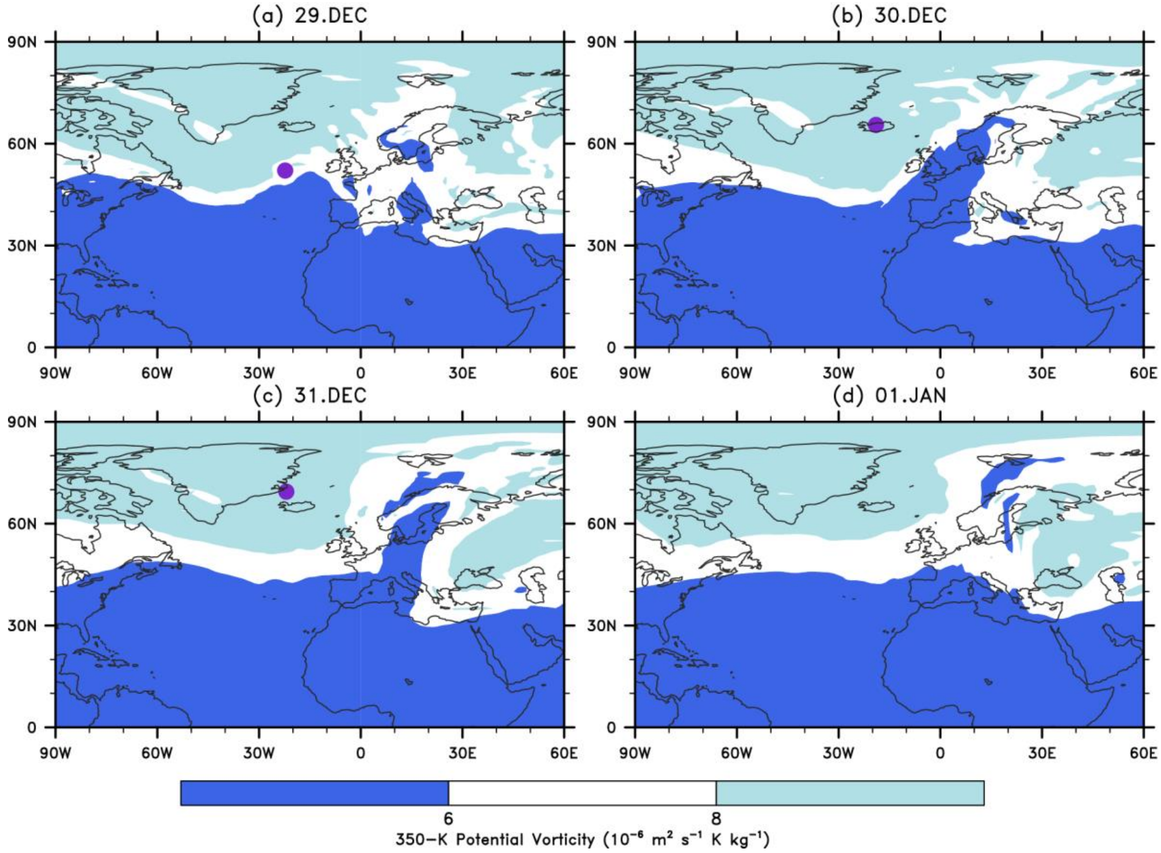

Figure S4. Temporal evolution of the potential vorticity map at 350 K from 29 December 2015 to 1 January 2016. The purple dot indicates the daily average center of Storm Frank. The NCAR Command Language (NCL) with version 6.3.0 (http://dx.doi.org/10.5065/D6WD3XH5) was used to generate the maps in this figure.

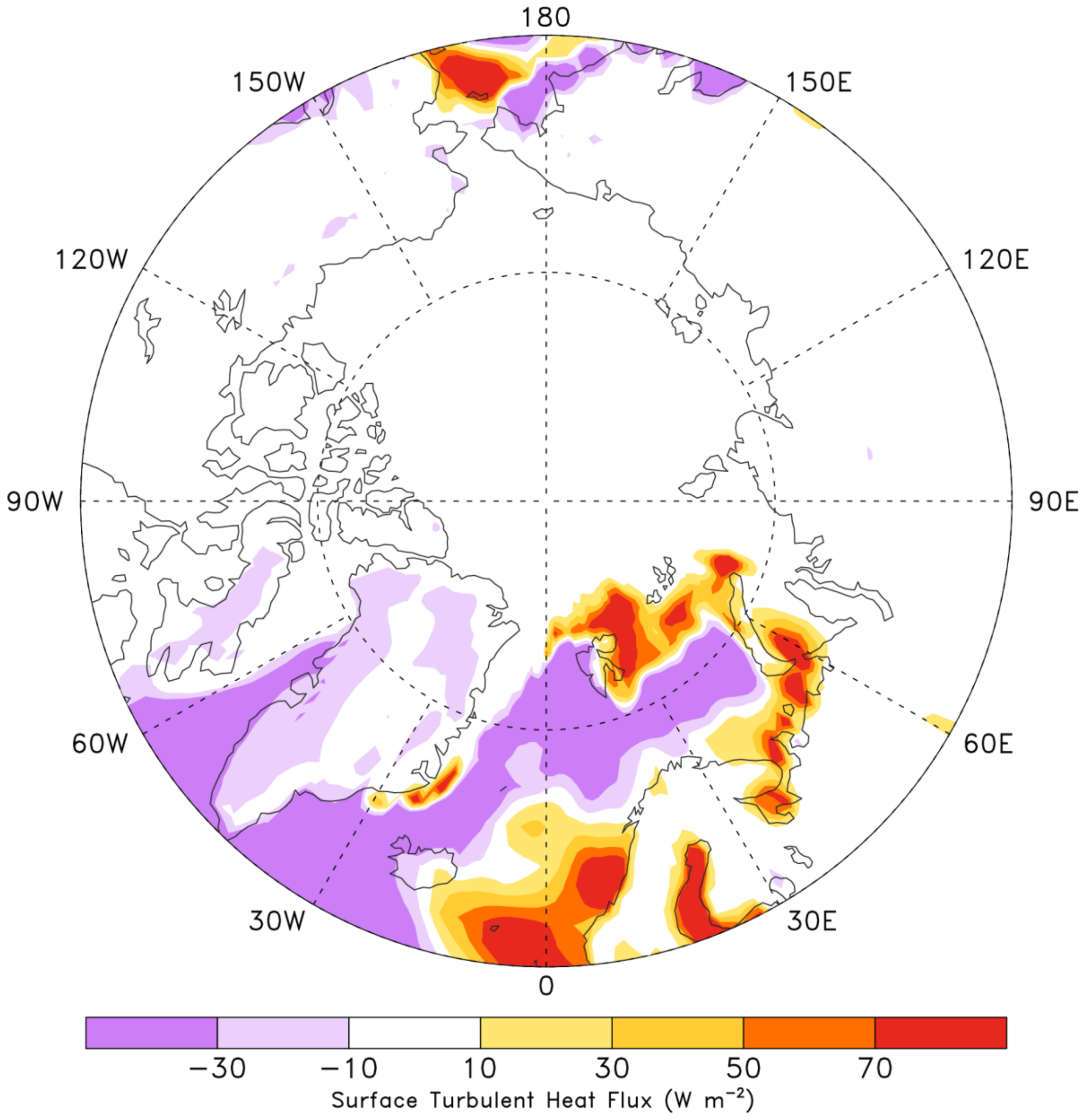

Figure S5. 20-day (1 to 20 January) averaged surface turbulent heat flux anomalies after the termination of Storm Frank (sensible plus latent; upward positive). The NCAR Command Language (NCL) with version 6.3.0 (http://dx.doi.org/10.5065/D6WD3XH5) was used to generate the map in this figure.

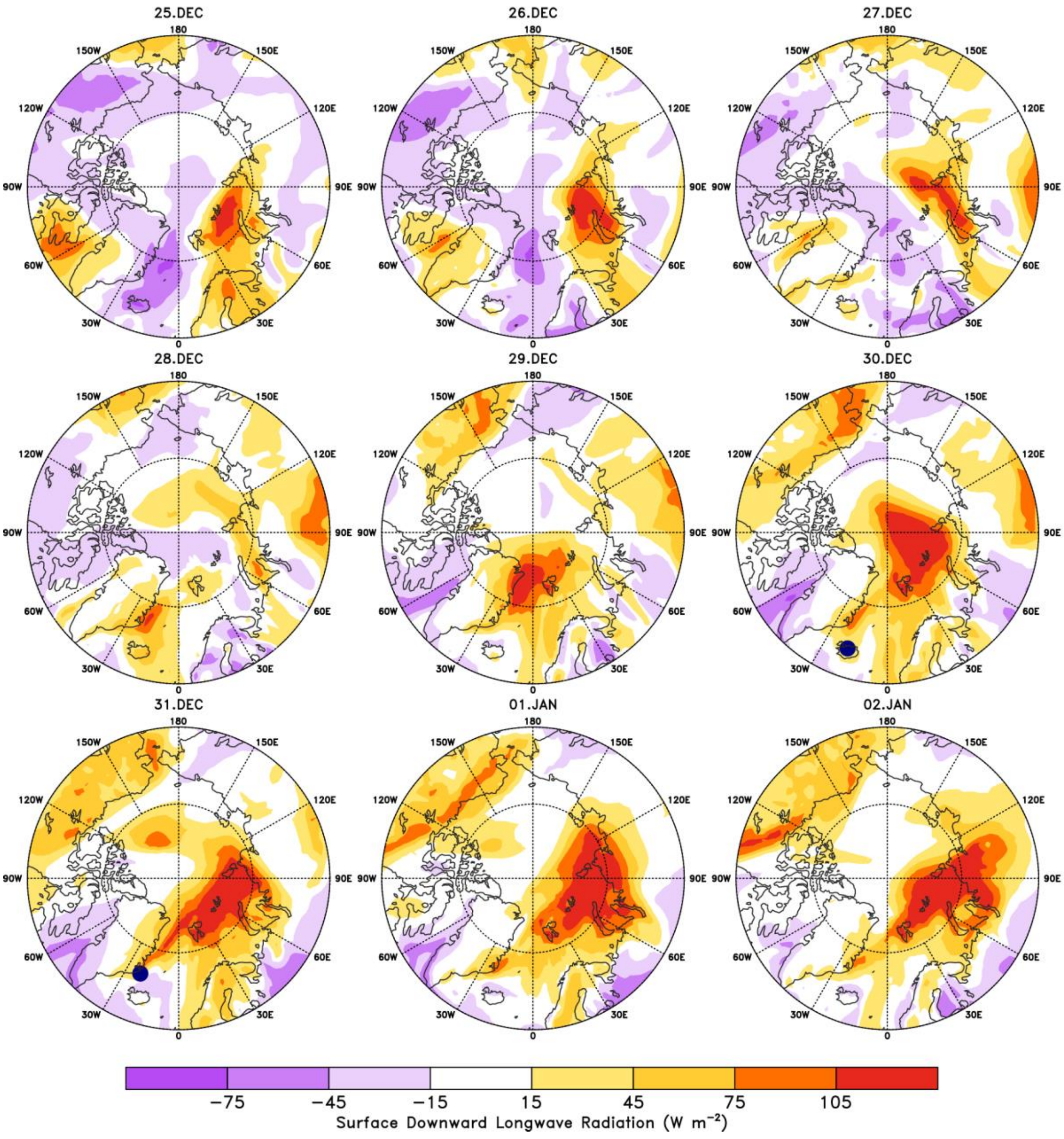

Figure S6. Same as Fig. S2, except for the surface downward longwave radiation (downward positive). The NCAR Command Language (NCL) with version 6.3.0 (http://dx.doi.org/10.5065/D6WD3XH5) was used to generate the maps in this figure.

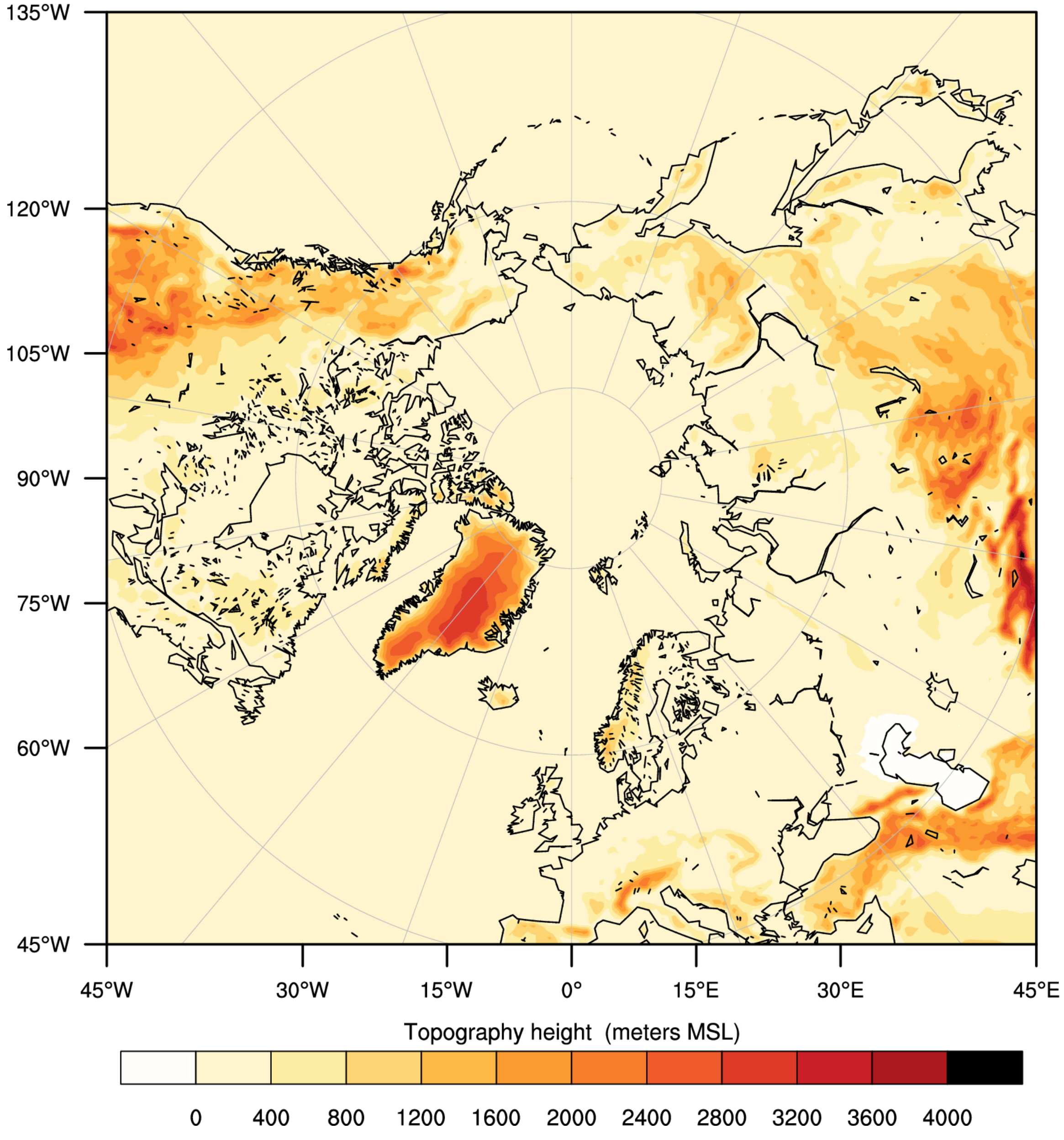

Figure S7. WRF domain for the sea ice sensitivity experiments. The shading denotes the height of the topography on 36 km horizontal resolution. The NCAR Command Language (NCL) with version 6.3.0 (http://dx.doi.org/10.5065/D6WD3XH5) was used to generate the map in this figure.

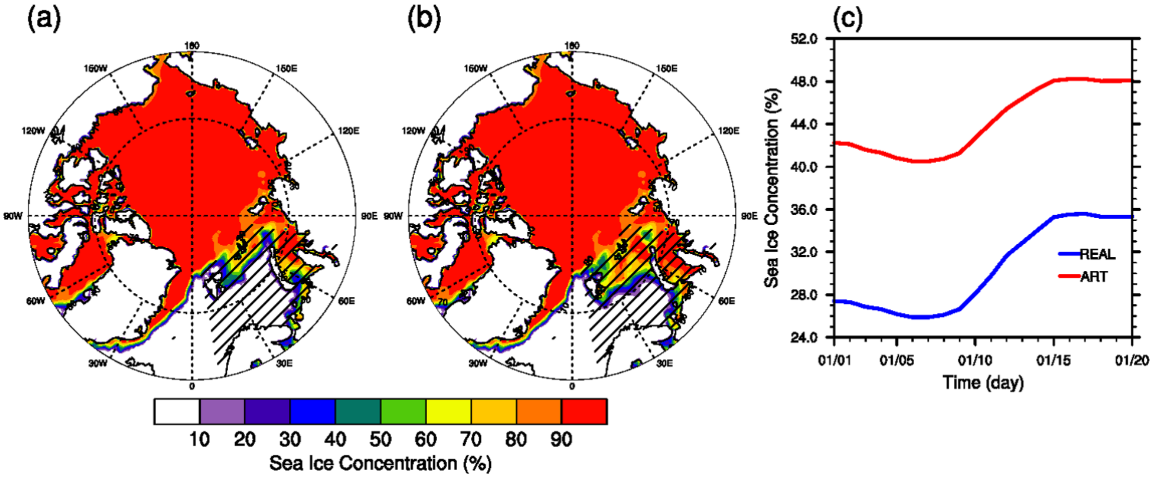

Figure S8. Maps of the sea ice boundary conditions averaged for 1–20 January and the area-mean sea ice concentration over the Barents and Kara seas. (a) real sea ice concentration and (b) artificially increased sea ice concentration (refer to Methods). (c) The area-mean time series of sea ice concentration (blue: real, red: artificially increased) over the domain of the Barents and Kara seas (hatched in (a) and (b)). The NCAR Command Language (NCL) with version 6.3.0 (http://dx.doi.org/10.5065/D6WD3XH5) was used to generate the maps in this figure.
